# An Automatic Weighting System for Wild Animals Based in an Artificial Neural Network: How to Weigh Wild Animals without Causing Stress

**DOI:** 10.3390/s130302862

**Published:** 2013-02-28

**Authors:** Diego Francisco Larios, Carlos Rodríguez, Julio Barbancho, Manuel Baena, Miguel Ángel Leal, Jesús Marín, Carlos León, Javier Bustamante

**Affiliations:** 1 Department of Electronic Technology, University of Seville, 41011 Seville, Spain; E-Mails: jbarbancho@us.es (J.B.); maleal@us.es (M.A.L.); cleon@us.es (C.L.); 2 Department of Wetland Ecology, Estación Biológica de Doñana (EBD-CSIC), 41092 Seville, Spain; E-Mails: carlos_r@ebd.csic.es (C.R.); jesusmarin@ebd.csic.es (J.M.); jbustamante@ebd.csic.es (J.B.); 3 ICTS, Singular Scientific and Technological Infrastructure, Estación Biológica de Doñana (EBD-CSIC), 41092 Seville, Spain; E-Mail: manuel.baena@ebd.csic.es

**Keywords:** sensor network, habitat monitoring, neural networks, computational intelligence

## Abstract

This paper proposes a novel and autonomous weighing system for wild animals. It allows evaluating changes in the body weight of animals in their natural environment without causing stress. The proposed system comprises a smart scale designed to estimate individual body weights and their temporal evolution in a bird colony. The system is based on computational intelligence, and offers valuable large amount of data to evaluate the relationship between long-term changes in the behavior of individuals and global change. The real deployment of this system has been for monitoring a breeding colony of lesser kestrels (*Falco naumanni*) in southern Spain. The results show that it is possible to monitor individual weight changes during the breeding season and to compare the weight evolution in males and females.

## 1. Introduction

The observation of animal behavior helps to understand key issues in ecology, such as which factors influence lifetime reproductive success [1], how parent–offspring conflicts are solved [2,3], which are evolutionary stable strategies [4] or which are the sex roles in reproduction [5]. This kind of study requires a large amount of data that are hard to obtain using classic methods of animal monitoring, because it is necessary to capture individuals repeatedly over short periods of time, which limits the amount and quality of data that can be obtained (captures alter individual behavior and may jeopardize the survival of the offspring, among others).

This paper demonstrates the performance of a remote monitoring system that can be applied to study animal behavior in some species. The system aims to bridge the abovementioned logistical and ethical gaps, thus allowing us to get enough data to interpret animal behavior.

Currently, we are applying the proposed technique in a prototype deployment to study a colony of lesser kestrels, a small insectivorous falcon. The study is based on the use of smart nest boxes that allow us to monitor several aspects of bird ecology. This system can provide all the acquired and processed data via the internet to scientists all over the World.

The use of modern technology to monitor bird behavior is not new. Several studies have already proven the utility of automatic monitoring systems able to deal with animal identification and body mass variation [6,7]. Some weight monitoring systems use a scale under the nest, but this cannot be applied to a species like the lesser kestrel that does not maintain a constant incubation area. Lesser kestrels do not build cup-shaped nests, but rather place the clutch on the surface of a cavity, crevice, or nest box. In addition, they move the eggs as they take turns or simply change the incubating position. Also, because of the hole-nesting habits, perching-based deployments [8] are difficult to implement. We propose the use of a smart nest-box, based on a widely used nesting structure for hole-nesting birds. This nest-box is designed to have low power consumption, and use low bandwidth wireless communications. This way, it can be easily deployed in areas with energy constraints. It is designed with a corridor in a way that forces the individual to pass through this area every time it enters or leaves the nest. The corridor is designed to obtain valuable information of the specimen, such as individual identification using RFID tags or its weight. Additionally, it allows adding easily extra sensors, if needed, to record as example humidity or temperature at the nest.

One novel aspect of our approach is the optimization of data labeling regarding stable and unstable measures. Animal movement on the scale causes a high proportion of unstable measurements (*i.e*., non-constant measures) according with the scale-implemented labeling protocol. In the lab these unstable measurements are discarded, but in the wild, this implies losing high amounts of data.

Other authors take this problem into account. For example [8] with a perching-based deployment consider the use of the maximum weight recorded as the most accurate estimate of body weight in its deployment but recognizes that for other balances, other arrangements of the weighing platform, or heavier animals, it might be necessary to use a more complex algorithm to convert recorded weight readings to accurate weight estimates.

Considering this, this paper proposes an Artificial Neural Network algorithm (ANN) that allows estimating a weight value from the acquired unstable values of a weighing event. Because of this, our system cannot only obtain patterns of body mass variation throughout the breeding period, but also allows for studying intra-day weight fluctuations. This permits detecting food provisioning events and their quality in terms of prey weight.

The rest of the paper is organized as follows: Section 2 focuses on animal condition monitoring issues. Section 3 describes the proposed monitoring system. A case study is described in Section 4. The results obtained with our system are shown in Section 5. Finally, Section 6 sums up the conclusions and presents final remarks.

## 2. Monitoring Animal Behavior Without Causing Stress

One important area of research is the study of animal behavior in the wild. Traditionally, most of the information about the lesser kestrel's behavior is obtained by direct observation from a distance with telescopes or binoculars, while the body condition of the birds is known via periodic captures of individual birds.

To obtain conclusions about individual behavior, it is necessary to gather large amounts of data, and this normally implies many years of field work because it is not possible to capture animals too often. First, the animals avoid being captured, and secondly, too frequent captures may stress the individuals, influencing their condition and behavior, also reducing the chances of offspring survival if they are breeding.

For this reason, we are proposing an automatic system focused on monitoring the condition of wild animals without causing stress, and therefore, without modifying their behavior.

The designed system must collect data without human interference in order to not disturb the animals, and must have reduced power consumption, operating in areas without electricity by using alternative energy, such as solar panels.

To estimate body conditions of animals, zoologist normally use body mass corrected by body size [9], Other methods based on the amount of subcutaneous fat [10], coloration of feathers or skin [11], or blood chemistry parameters [12] have also been used. However, they generally require the capture of individuals, and therefore, they are not suitable for automatic analysis without human intervention. In this study, we have focused on the analysis of body weight. Weight is a good indicator of the individual's condition (animals in good condition normally have a body weight within a certain range around the optimal weight of the species). Despite the fact that corrections due to differences in body size among individuals would improve the suitability of this measure, individual body mass and its deviation from the gender-average is informative about its body condition. In addition, individualized data provided by RFID tags allows the consideration of individual ID in the analyses circumventing biases due to differences in body size among individuals. But for some species, such as the lesser kestrel, there are no big variations in size amongst individuals of the same gender.

We obtained individual body mass by placing the scale in an area where the animal had to pass through. This passage area must be chosen in relation to the species' habits. For species breeding in nest boxes, the entry of the nest box is a good place—the animal often enters several times a day and, during the breeding season, it allows us to estimate the number of foraging bouts parents make to feed the offspring. All these data are valuable for zoologists, and allows them to obtain large amounts of data within a short time.

Behavioral monitoring has evolved greatly due to the boom in sensor network technology. The increase in the use of sensor networks has had several consequences—first, the quality of data grows over time and on the spatial domain; second, the possibility of transmitting the measured data through the network increases the need of having high bandwidth communications; and third, the reduction in the cost of the data storage makes it possible to store large amounts of data.

All these consequences also imply some negative effects—an increase in data traffic, an increase in data stored, and an increase in power consumption. Some authors have taken these effects into account [13,14] and have expressed the need to employ processing techniques in order to circumvent these handicaps.

There are different approaches for behavioral monitoring. Some of them use wireless sensor network technology in order to acquire and process the physical data [15–18]. Others focus on the required middleware that allows access to the physical data [19,20].

The proposed system takes these issues into account and consists of a smart scale system, strategically deployed and hidden at the entry of the nest area of the animal to be monitored. It allows acquisition and automatic processing of the sensor signals, communicating valuable data to researchers anywhere in the world.

## 3. Obtaining Stable Measurements Using a Dynamic Weighting System

Most common weight acquisition systems are not suitable to acquire data about dynamic targets. In general, they only offer a measurement when the estimation is stable, that is, when the oscillation between consecutive measurements are below the accuracy of the device.

In a dynamic system (*i.e*., a system where the object to be measured does not remain static), the above-mentioned factor does not apply. In addition, if the system requires that the animal is not stressed, it is difficult to keep the animal in a static position for a long time. Moreover, the random movements of animals do not allow the system to obtain regular weighing patterns (the set of measurements obtained while the animal is on the scale).

To improve these results, a novel smart scale system is proposed. It allows obtaining weight estimations from both stable and unstable measurements. This algorithm has been designed to fulfill the following goals:

- To reduce the amount of useless data using local pre-processing
- To increase the accuracy of the measurements (around 5 g in our case study)
- To increase the amount of useful data, estimating a body weight from patterns with non-stable measurements
- To allow its execution on devices with low resources

**Figure 1.**
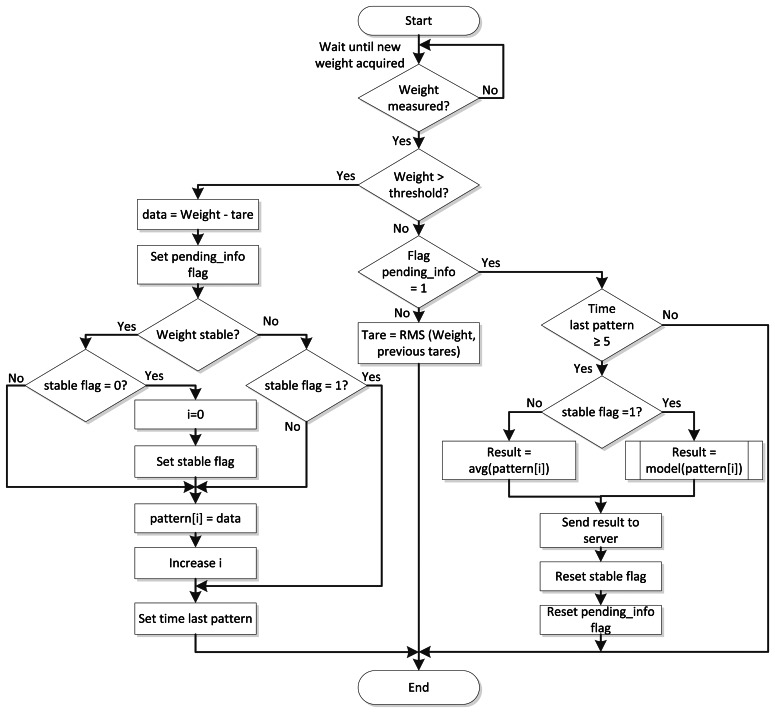
Flow chart of the weighing algorithm.

The proposed system allows increasing the accuracy of the measurements using a tare calibration. This calibration is taken from the weight measurements obtained when there is nothing on the scale pan. The algorithm used to increase the amount of useful data is based on a computational intelligence algorithm. The algorithm used to estimate weight over the dynamic system is summed up in Figure 1.

This algorithm would be implemented over devices with low resources, such as the microcontroller used for controlling the smart nest boxes. The next section describes the main tasks undertaken for development by the proposed weight acquisition system.

### 3.1. Acquiring Weight Data

There are many scales, based on different kind of sensors, such as piezoelectric sensors, strain gauge, *etc.* Some commercial devices, especially designed for laboratory use, can fulfill the required specification for the scale, but in general, these scales require that the object whose weight is to be acquired remain static on the scale pan.

There are some scales that send raw data of the acquired weight. They periodically send the estimated weight plus some additional information, such as if the acquired weight is considered stable or unstable. This is obtained using as criteria whether the estimated weight within a series has a variation above or below the accuracy of the system. In our case, we proposed the use of one of these devices. We used a scale that offers 16 raw measurement per second, tagged automatically as stable or unstable.

In a traditional weight measurement system, only stable measurements are considered, discarding measurements that are not stable. But in our test, it is infrequent that the animal remains long enough on the scale for the system to obtain stable measurements (in our estimation, it occurs only one out of 10 measurements, around one out of 10 acquired patterns). This paper proposes using all the acquired weight values, trying to estimate a stable weight from unstable patterns, increasing the amount of useful data.

### 3.2. Previous Treatment: Weight Preprocessing

Preprocessing the data aims to increase the accuracy of the measurement and reduces outliers.

#### 3.2.1. Reducing Outliers

In some situations, the scale may offer erroneous values that can affect the acquired results. For example, the wind can produce weight fluctuations recorded by the scale.

It is possible to filter these values by considering an expected acquired weight range. In the proposed case, lesser kestrels have a body weight in the 100–190 g range. Using this information, we consider an extended 50–300 g range to ensure a correct acquisition of the weight dynamics due to the movement of the animal.

Additional outliers can be avoided by evaluating the behavior of the pattern. If we consider a pattern wherein an animal remains on the scale, it is possible to see that there is a minimum amount of time required for the animal to cross the system. It depends on the dimension of the pan and the animal species.

In our case, only patterns longer than a second are considered, that is, patterns with more than 16 weight measurements, considering the frequency of measurements recorded by the scale used. Weight patterns with a lower number of measurements were discarded.

#### 3.2.2. Increasing Weight Accuracy

To increase the accuracy, a tare calibration is necessary. In our test, we have seen that the tare changes slightly during a year, due to dirt accumulated on the scale pan. Obtaining measurements with high accuracy, in the order of grams, requires taking into account these variations. Measuring the tare once a day can offer the required accuracy. Our system continuously updates the tare when there is no animal over the pan, fulfilling this requisite.

This calibration is obtained by consulting the weight measured by the scale when there is no animal on the pan. During this time, the system updates the tare using an iteratively reweighted least squares method (IRLS). To obtain the calibrated weight data, the body weight estimation of the pattern is updated with this tare.

A calibration of the gain of the scale is done once per year. This calibration is done with several calibrated weights, to ensure the accuracy and linearity of the stable measurements weighing in static conditions. Scales were also tested against the one used for classic field work before their final deployment, offering deviations below 1 g for the same individual, which we found suitable for our study. In addition, we also captured individuals with different purposes during the breeding season, and the small differences between automatic and manual weights remained constant.

### 3.3. Pattern Acquisition

We consider as a pattern the set of measurements obtained from the time the animal steps on to the scale pan until its departure. To obtain this, the system should distinguish these two events. The system knows when an animal arrives on the pan because of the sudden increase in the measured weight. When the scale detects a calibrated weight over a defined threshold, it starts to acquire the pattern and leaves the auto-calibration process.

This threshold can be obtained as a function of the body weight of the animals to be monitored. In our deployment for the lesser kestrel (with a body weight range of 100–190 g), a threshold of 50 g to start captures has been used.

During the entire pattern acquisition, the system stores the calibrated weight and stability data gathered from the scale in an array for its processing.

To detect when an animal leaves the pan, the same threshold is used, but in this case, it is necessary to obtain more than four weights below the threshold. At that moment, the system stops acquiring measurements, removing the last four measurements below the threshold. Valid patterns, without outliers and with duration higher than the minimum, are sent for the main processing stage.

### 3.4. Main Processing

The main processing task is used to estimate the weight of an animal. It can be divided into two different stages; offline processing, used in a PC to model the algorithms, and online processing, implemented on a low power consumption system.

#### 3.4.1. Offline Processing

Offline processing is required to model the proposed algorithms. It was done only once, before the implementation of the system, using historical data.

It is important to consider that weight can be easily estimated on patterns with stable measurements. But according to our test, only 15% of the measured patterns had stable measurements. This fact considerably reduces the amount of valid data gathered from the system. To avoid this issue, a machine learning algorithm is proposed in order to obtain weight estimations from non-stable weight patterns.

Machine learning is a branch of computational intelligence concerned with the design and development of algorithms that can recognize complex patterns, and make intelligent decisions based on the input data. These algorithms allow the discovery of knowledge from specific input data and experiences, based on statistical and computational principles, and generalize the results of unknown situations.

Machine learning is applied to retrieve weight estimation from unstable patterns. Machine learning is widely used in pattern recognition [21]. Several authors have applied these techniques monitoring in nature, especially in the environmental monitoring area [22–24]. The use of machine learning for animal monitoring or control is less common, but some authors have used it to estimate habitat selection of birds [25] or fish [26].

Non-supervised computational intelligence techniques, such as self-organized maps (SOM) [27] or Vector Quantization (VQ) are not suitable for our application because we have some stable measurements that allow us to train the system.

Classification supervised computational intelligence techniques, such as Support Vector Machine classifiers [28] were also discarded due to the need of a system with the ability to be used as an arbitrary function approximation mechanism that learns from observed data. Examples of machine learning techniques that can approximate a function using training data are Artificial Neuron-Fuzzy Inference Systems (ANFIS) [29], Case-Based Reasoning expert system (CBR), Support Vector Regression (SVR) or Artificial Neuronal Networks (ANN). ANFIS has many applications in the evaluation of complex systems, but it requires a previous knowledge of the system to design the rules and the initial system. This system was discarded due to the complex forms of the patterns that do not easily allow acquisition of this initial system.

Expert Systems or case-based expert systems were not considered as the amount of previous data gathered from the smart nest box was insufficient.

Both SVR and ANN are suitable to implement to obtain a function that approximate the weight estimation. Both techniques are tested, using as input variables are the following ones, obtained from the input pattern:

- Max_1: The most repeated weight in a pattern (the largest, if multiple)
- N_1: Number of repetitions of the previous variable in a pattern
- Max_2: The second most repeated weight in a pattern (the largest, if multiple)
- N_2: Number of repetitions in a pattern, of the previous variable
- Max_C1: The most consecutively repeated weight in a pattern
- NC_1: Number of repetitions of the previous variable in a pattern
- Max_C2: The second most repeated weight, consecutively, in a pattern
- NC_2: Number of repetitions in a pattern, of the previous variable
- N_EL: Total number of weight measures in a pattern

These variables allow characterizing the weight pattern, without complex calculations. It allows implementing the weight pattern directly over low resource devices. ANN and SVR models require a learning process that is executed only one time in offline processing. In this case, this learning is done with a back-propagation algorithm for ANN and an epsilon-SVR parameter optimization for SVR.

The training set and validation set are obtained from patterns with both stable and unstable measurements obtained in the first year of operation of the system. During the first year, all raw weight measurements were stored in the central server.

The target of patterns is defined as the arithmetic mean of the stable weights of the pattern. We are considering the mean stable measurement of a pattern as the target for training the neural network, relying on the ability of the scale of getting an unbiased weight estimate when values are tagged as stable. The rest of the parameters are obtained from the input pattern after removing all the stable measurements.

These training sessions are done with 50% of the 7,856 available patterns with stable weights, that is, with 3,928 patterns chosen randomly from the total available. The other 50% were used for testing. The PC used to train the network requires less than two minutes training and evaluating every one of the tested models.

**Figure 2.**
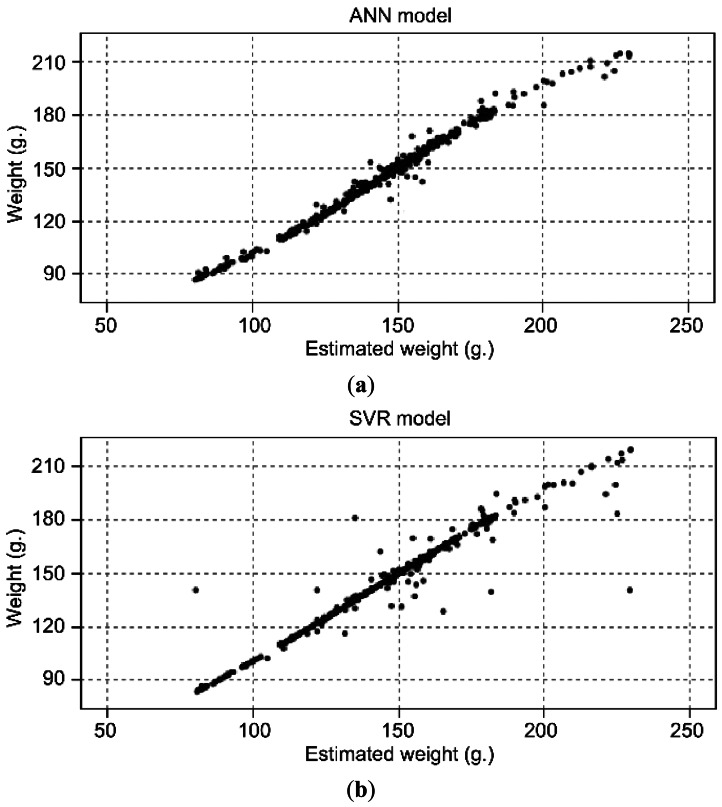
Comparison between real weight and estimated weight. (**a**) ANN model; (**b**) SVR model.

Both techniques (ANN and SVN) offer good accuracy, according to the results obtained with the validation set. 98.5% of accuracy with ANN, in comparison with a 97.3% of accuracy with an AVR model, using the same input and validation set for both models. This accuracy comparison is done with the best models obtained with the two techniques. Figure 2 depicted a comparison between real and estimated weight using ANN and SVR models.

As it can be seen, for the proposed application ANN accuracy is slightly better. Due to this, the Artificial Neural Network model was finally chosen. Moreover, ANN models are widely used to approximate unknown models as a function from observations in complex systems, such as modeling human behavior [30] or detecting machine faults [31].

**Figure 3.**
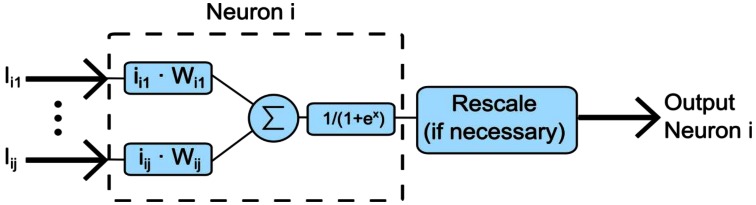
Artificial neuron of a multilayer perceptron network.

Different neural networks were evaluated. Finally, a multilayer perceptron (MLP) model was chosen. The multilayer perceptron network consists of multiple layers of neurons, each one with a sigmoid transfer function. Every neuron is weighed and connected to all the neurons of the previous layer. This is depicted in Figure 3, where *W_ij_* is a multiplier factor that represents the importance of the input *j*.

**Figure 4.**
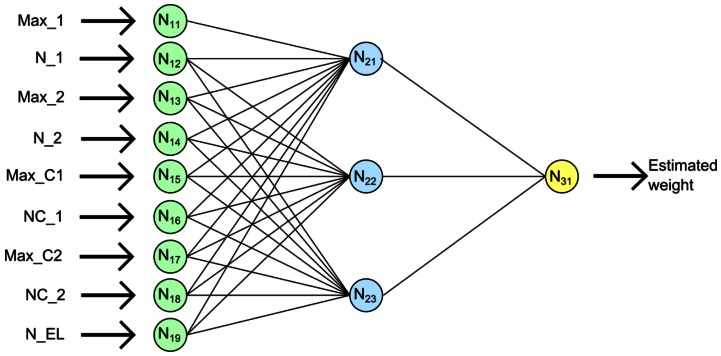
Proposed MLP network.

In this case, the proposed network has a structure of nine neurons in the input layer (one per input), three neurons in one hidden layer, and one neuron in the output layer. The output neuron offers as output an estimation of the weight of the studied animal that produces the evaluated pattern. This neural network is depicted in Figure 4.

Using this information, different MLP networks were tested, with from 1 to 9 neurons in the hidden layer. Finally, the proposed MLP with three neurons in the hidden layer was chosen. This is because this model offers the best trade-off found between accuracy and complexity of the network. This system is designed to be used over devices with low resources which do not allow the use of complex models. Table 1 sums-up the connection weight of the proposed MLP network, where neuron *N_i,j_* represents the neuron *j* of the layer *i*, according to the Figure 4. From *N_i-1,j_* represents the connection between neuron *j* of the layer *i-1* and neuron *N_i,j_*.

These connection weights are rounded out to six decimal. These weights are obtained by training for its use to monitor the Lesser Kestrel colony with the proposed smart-nest boxes.

#### 3.4.1. Online Processing

The online processing is executed in every nest box with each new acquired weight pattern. Two different algorithms are used, depending on whether the pattern has or does not have stable measurements. In case it has stable measurements, the weight of the animal is obtained as the arithmetic means of these stable measurements. It can be obtained by executing the following pseudo-code:
Sum := 0num := 0for i in 1 to Nsamples ; for each data of the pattern if (data[i] is stable)  sum := sum + data[i]  num := num + 1weight := sum/num

This algorithm does not require complex calculations, and can be executed over devices with low power resources. We consider that stable measurements are obtained in the same conditions as the gain calibration. For this reason a similar accuracy is expected. For the scale we use, this accuracy is better than a gram, several times higher than the required accuracy.

In the case the pattern does not have stable measurements, the weight is estimated by executing the online processing of the modeled neuronal network. The execution algorithm is presumed on the following pseudo-code:
for j in 1 to 3; each layer for k in 1 to Nj[j]; each neuron  sum := 0  for m in 1 to Nk[ij]; each input   sum := (sum + I[mkj]*W[mkj]   output[kj] := 1/(1 + eˆsum)weight := Resample_factor * output[output neuron]

This algorithm is useful to estimate the weight in a dynamic system, when the shape of the pattern is not predictable.

### 3.5. Sending and Tagging Useful Data

Initially, the system was designed to send all the acquired raw data (without any processing) to a base station. The system worked in this way during its first year of deployment. However, it caused high bandwidth consumption in the communications interface when all the deployed smart nest-boxes are sending raw data to the base station.

An important amount of the bandwidth used can be saved using only preprocessing, dealing locally with the calibration processing. Furthermore, sending entire patterns to a central database uses extra energy to store data that is not directly useful to researchers.

The proposed system reduces the amount of useless data, sending only one weight estimation of the studied animal each time it crosses the corridor. This is why this system has a low bandwidth requirement and can be further used in devices with low resources with low power consumption.

The estimated weight is sent to the main server, where it is stored with an associated tag, which identifies whether this weight is obtained from a pattern with stable weights or not. With this system, it is not necessary to send all the raw weight data to the base station, only the weight estimation, tagged as stable or unstable, saving data bandwidth.

Currently the system is programed in such a way that it is possible to configure each smart-nest box to send (or not to) the raw weight data of the pattern. In normal operations, it is sending only the weight estimation and its tags, but for test, diagnostic and verification, we can remotely configure each nest to send the raw data.

## 4. A Case Study: Lesser Kestrel Monitoring

**Figure 5.**
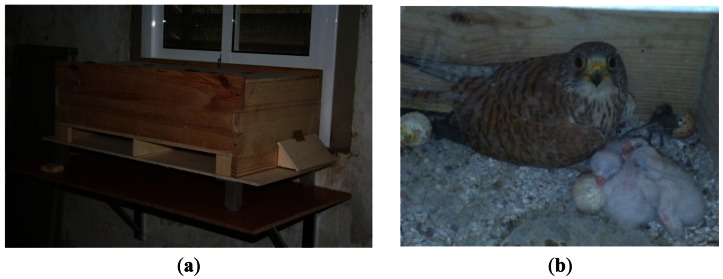
Smart nest boxes: (**a**) Exterior view; (**b**) Interior view.

The lesser kestrel (Figure 5(b)) is a small migratory falcon inhabiting open landscapes [32]. It is a colonial species that breeds in old buildings, such as churches or castles, within urban areas in Western Europe. The species experienced a significant decline in its Western Palearctic breeding range in the middle of the 20th century [32,33]. Previously considered as one of the most abundant raptors in Europe [34], the lesser kestrel became extinct in several countries (e.g., Austria, Hungary, and Poland) and practically disappeared in others [35] (e.g., France, Portugal, and Bulgaria).

Mediterranean Spain constitutes the lesser kestrel stronghold in the Western Palearctic [33]. However, the Spanish population also suffered a precipitous decline, as it dropped from an estimated 20,000–50,000 pairs in the 1970s [36] to some 4,000–5,000 breeding pairs in 1988 [36]. This decline has been caused by the reduction of both the extent and the quality of foraging habitats [37]. The species is also sensitive to climate change [38], making it a good model species to study the impact of global change on an endangered species.

### 4.1. Previous Studies

The “Estación Biológica de Doñana (EBD-CSIC)” has been monitoring lesser kestrel colonies since 1988. It has been recording colony occupation and breeding success in terms of the number of fledglings and the proportion of successful nests. On average, the birds monitored were captured (sightings of banded birds with telescopes are considered recaptures) three times in the same breeding season (range: 1–70). In approximately 45% of the cases, body weight was manually recorded and the maximum number of weight measurements per individual bird and year was four.

Using these measurements, important dates of the breeding period can be obtained [38], such as arrivals (10 February), median laying date (9 May), median hatching date (1 June) or median fledging date (7 July). But this frequency of measurement is insufficient to evaluate the individual weight trend, the pattern of body weight variation of breeding adults from arrival at the colony in mid-February to the end of the nestling period in mid-July is not well known. Likewise, intraday variation in the body weight is completely unknown.

### 4.2. Automatic Lesser Kestrel Monitoring

To monitor this species, and using the techniques described before, a prototype installation called HORUS was deployed. It is a distributed system deployed in the grain elevator of “La Palma del Condado” (“Huelva” Province, SW Spain). At this site, researchers of the “Estación Biológica de Doñana (EBD-CSIC)” have been studying the lesser kestrel colony since 1994. In this colony, kestrels nested on the windowsills of the grain elevator that were sheltered and sufficiently enclosed to make suitable nesting sites.

For the prototype installation, we selected the windows on the sixth floor of the building where smart nest boxes were installed and readily accepted by the kestrels during preliminary checking (three and four nest boxes during 2008 and 2009, respectively) and also when the definitive prototype installation was made in 2010 (20 nest boxes). The main constituents of the developed installation are briefly described in the next section.

#### 4.2.1. Smart Nest-Boxes

The smart nest boxes (Figure 5) are the main components of the monitoring system. They consist of the following two blocks:

- The nest cabinet
- The electronic scale

**Figure 6.**
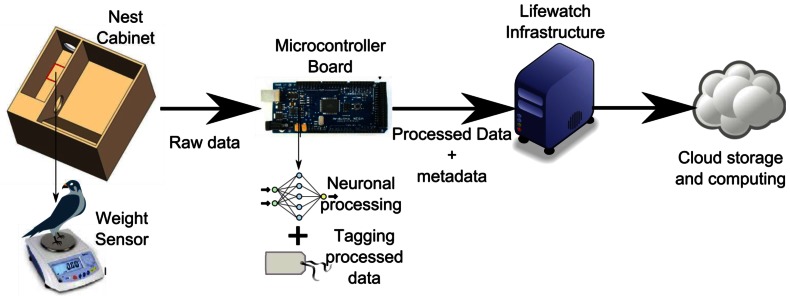
Smart nest architecture.

The nest cabinet (Figure 6) is divided into two parts—a corridor and the incubation chamber. This nest cabinet has a smart design to ensure that the birds pass the corridor each time they enter or leave the nest. It forces the birds to pass over the scale every time they enter or leave the nest and, therefore, ensures obtaining the measurement of the weight. The control of the scale sensor is done with an ATmega2560, an economic, low power, and robust microcontroller. It controls and processes the nest's sensor signal and sends it to the process server through a communication interface.

The program implemented in the microcontroller performs the following tasks:

- Identify the individuals using a RFID reader
- Obtains the tare of the scale
- Obtains the body weight estimation measurement from the digital scale
- Preprocesses all the weight data
- Sends the weight estimation to the central server

The used weight sensor sends up to 16 values per second to the microcontroller, which executes the weight preprocessing described in Section 3.

Apart from the electronic system used for automatic monitoring, each nest-box of the current prototype has an analog grayscale video camera connected to a network video server. This camera can be used to observe the nest content and can be programmed to record and store video data in a dedicated storage server. This video data allowed us to validate the conclusions obtained with the automatic monitoring system.

It is important to consider that the high energy consumption and high data bandwidth requisites of the video system would not allow its use in nest-boxes deployed in remote areas powered by solar panels. But removing the cameras, the rest of the system consumes very little power and data bandwidth. It allows its use with small solar panels of dimensions around 9 × 6 inches. All data sent to the central server are tagged by the microcontroller with additional data, such as a timestamp and the identifier of the nest. It allows us to classify the data in the central server for future analysis.

#### 4.2.2. Storage and Remote Access

The gathered data from all the nest-boxes is stored in the database of a main server. This main server is located in the “Estación Biológica de Doñana (EBD-CSIC)” and allows biologists all over the world to access easily the stored data with standard SQL queries. This stored data is tagged with localization and some identification codes. The database is designed to allow the integration of smart nest-boxes with different sensors.

Moreover, the system is designed to be integrated as a provider to LifeWatch infrastructure [39]. LifeWatch is a European project that aims to construct and bring into operation the facilities, hardware, software, and governance structures for all aspects of biodiversity research. With LifeWatch, researchers can access the nest data through web applications and e-labs, facilitating the task of consultation and data processing.

## 5. Experimental Results

The monitoring system has been working since 2010. During the first year, the system acquired all raw data without processing. These data were used in the neural network training. The main characteristic of the data gathered in the database is summarized in Table 2.

Using the proposed system, 56.21% of the patterns without stable measurements could be recovered. Without the proposed algorithm, all these patterns would have been discarded.

To summarize, the local processing allows us to drastically increase the amount of valid data gathered by the system, reducing at the same time, the used throughput of the network, the collisions, and the energy consumed in sending the captured data to the central server.

### 5.1. Network Performance

A year after deployment, the analysis of the data allowed us to detect some network conflicts with the initial deployment. For example, if different nest boxes acquire weights from individuals at the same time, they compete for control of the bus, causing data collisions and delays in transmitting data. The proposed intelligent weight estimation system allows avoidance of these network collisions because it reduces drastically the amount of data sent to the base station. It is possible to compare the data traffic using the proposed data fusion algorithm with the gathered data during the first year of deployment, when all data are sent to the base station.

Knowing that the scale offers 16 samples per second (*SPS*), the average payload of the application layer per pattern of the system without data fusion can be obtained with the Equation (1):

(1) $$N_{T,\text{raw},\text{no agregation}}=16\cdot \text{SPS}\cdot P_{T}\cdot N_{\text{Bytes}}\cdot N_{\text{msg}}$$

where *N_T,raw_* is the number of bytes to send per day at the application layer; *P_T_* is the length of the pattern in seconds; *N_Bytes_* is the number of bytes to send (16 bytes in this case), and *N_msg_* is the number of messages per day.

However, with the proposed algorithm, only one message per pattern is sent. In this case, the payload per pattern can be obtained according to Equation (2):

(2) $$N_{T,\text{raw},\text{agregated}}=N_{\text{Bytes}}\cdot N_{\text{msg}}$$

As can be seen, the proposed algorithm permits reduction in the number of bytes sent to the base station to the order of 16 · *SPS* · *P_T_*. Considering the 23.18 s of the average pattern, a total amount of 371 bytes per pattern can be saved to send to the base station using the proposed aggregation. It is especially important in the case of the usage of low-power area networks transceiver, such as 802.15.4 low-rate wireless transceivers.

For example, using a CC2420 radio transceiver—widely used in wireless sensor networks devices—allows a saving of 99.76% of the energy used in data transmissions, considering a power consumption of 38 mW in the transmission mode [40].

These results are summed up in Table 3 [41], considering a TelosB node. They depict the average consumption of a pattern with or without data aggregation. This result considers the average pattern length of 23.18 s and the energy consumed by the microcontroller with the data aggregation algorithm.

To conclude, the local processing allows us to drastically reduce the used throughput of the network and the energy used in data transmission, especially on days with a high number of patterns. The reduced power and data bandwidth consumption of the smart-nest box (without using the video cameras survey system) allow it to be used in remote localization, with constrained energy available. Moreover the proposed local processing allows us to reduce the unnecessary and redundant data sent to the data server. It not only reduces the power consumption, but facilitates the access and analysis of the stored data.

### 5.2. Body Weight Estimation Accuracy

With the evaluation set (the 50% of patterns acquired during the first year and not used for training), the system offers an accuracy of 98.5%, that is, an error lower of 4 g. in relation with the mean weight of the birds.

Table 4 sums up a comparison between the weight measures obtained by hand (*i.e*., capturing the kestrel) and the nearest (in time) weight estimation obtained by the system for the same individual. The weight estimation of the smart nest-box was obtained before or after the recapture, when the individual returned to the nest. All these weights were obtained from unstable patterns.

In our results, stable patterns, due to the tare and gain calibration, offer weight estimation with accuracy better than a gram. As can be seen, the system offers an error in the range predicted by the evaluation test. The proposed system has an accuracy of up to 4 grams. This accuracy allows the analysis of weight time series for the evaluation of temporal changes in individual body weight. Sometimes, it even allowed us to estimate the weight of the prey brought to the nest, especially when medium-sized prey were brought to the nest to feed the nestlings.

### 5.3. Analysis of the Weight Evolution during a Day

**Figure 7.**
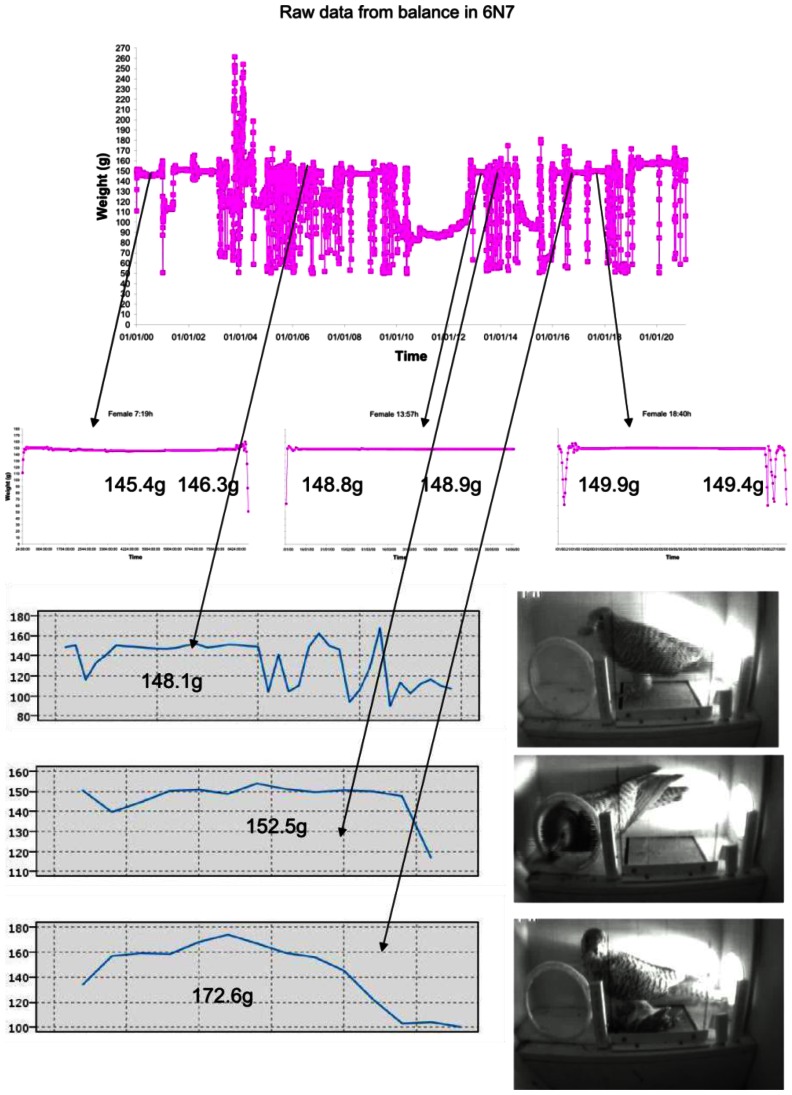
Different weight patterns of kestrels with prey shown with the video recorded simultaneously.

The lesser kestrel mainly feeds on insects, but sometimes can catch slightly bigger prey, such as small rodents, birds, or lizards (weighing around a dozen grams). During a real test (Figure 7), the system demonstrated having enough accuracy to acquire the weight variations caused by the prey, with both stable and unstable measurements. Moreover, the proposed system allows for an analysis of the frequency of big prey captures. As can be seen, the system can detect the difference of weight produced by the prey (even with unstable patterns). It allows researchers to obtain important information about the foraging habits of the observed individuals.

### 5.4. Analysis of the Weight Evolution of the Colony during the Breeding Period

**Figure 8.**
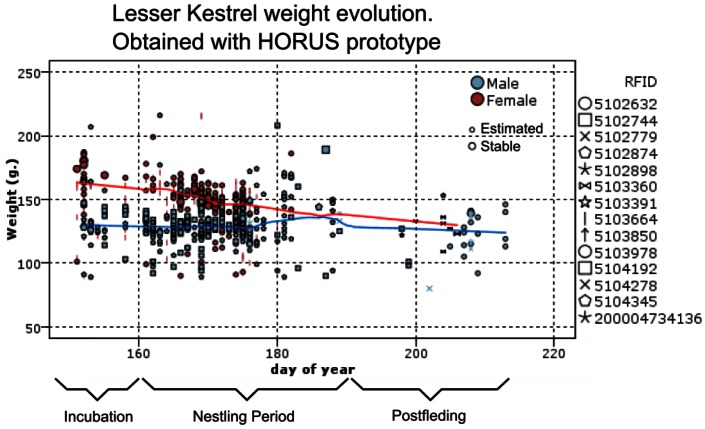
Weight evolution obtained with the proposed system from different nests.

The proposed system allows monitoring the evolution of body weight of the animals during the breeding season. Figure 8 shows these results acquired during the year 2010 with the proposed system. This figure shows the data acquired from the system during this year. Due to an error in the storage server, we not have data in 2010 before the first of June.

In this figure, each symbol represent a different animal witch its RFID code, from 14 tagged lesser kestler. Big symbols represent weight obtained from stable measurements and Small symbols (the majority) are estimation from unstable patterns. Red symbols are measurements from females and blue ones are from males. The Y-axis represents the day of the year, being 1 the first of January. According of the information described in Section 4.1, most important events during the breeding season [38] are highlighted in the figure.

The colored lines represent the seasonal trend, blue one for males and red one for females. As it can be seen, this species has an important body mass variation during the breeding season that this system allows us to study with only one-year data, circumventing limitations posed by different feeding conditions among study years.

**Figure 9.**
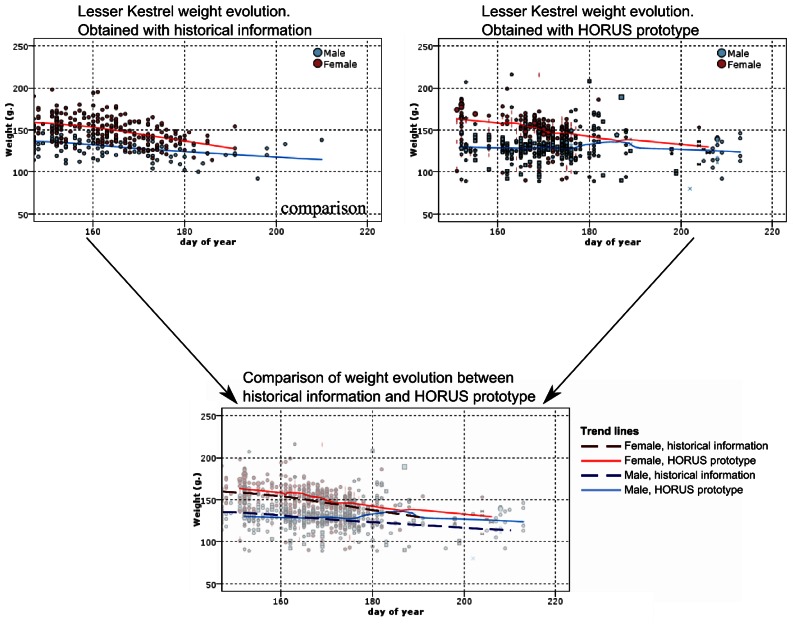
Weight evolution obtained on the basis of manual measurements along several years of monitoring. Each point represents a single measurement of an individual.

Obtaining similar data by recapturing the individuals requires a lot of time and effort. For example, Figure 9 shows a comparison of the body weight evolution, using the proposed system *versus* historical data from manual captures. Historical data took 24 years (1988–2012 breeding periods) for the acquisition of data with sporadic captures of different individuals. Different years of manual captures are overlaid.

As it can be seen, the results obtained from the historical data are similar to the ones obtained with the proposed prototype. The HORUS prototype offers in a year similar data to that obtained in 24 years of manual captures. The proposed system would allow the researchers to estimate the cost of breeding in terms of bodyweight loss and the evolution of this parameter during the breeding cycle. This cost is directly associated with the foraging trips to feed the nestlings. The system allows quantifying the number of trips, its duration, and the weight loss by the parents during the trips. With our system, it is possible to measure variations body weight trends among years. Due to the limited acquired data, the manual capturing system does not allow conducting this kind of analysis.

### 5.5. Qualitative Results of the Proposed System

Despite it is being difficult to estimate the stress caused by the monitoring system to the animals; the quantifiable results obtained suggest a low impact of the system on the animals:

- After system deployment (breeding seasons of 2011 and 2012), almost all nest-boxes were visited and used for sleeping during the arrival and mate acquisition periods. A high proportion of them were indeed used for breeding (15 out of 20, and 11 out of 20, in 2011 and 2012 respectively). This suggests that neither nest-boxes nor their equipment were hostile to lesser kestrel.
- Breeding success of breeding pairs was around 2–3 fledglings per nest, which was similar to the breeding success recorded in natural colonies in the surroundings.

## 6. Conclusions

The main goal of the proposed system is to use current technological advances in a real-world application in the area of biodiversity conservation to study how global change could affect a colonial and endangered bird species. With the results obtained we concluded that the proposed system would use low resources and be of low cost.

The prototype deployed in Spain for evaluation with the lesser kestrel has demonstrated to be a good method to study these animals. The proposed system allows us to carry out this evaluation without stressing the animals and without the need of a human observer. As a consequence, this monitoring does not influence the animal behavior, offering reliable data to researchers that can be accessed in real time through the internet.

The authors are currently working on several improvements of the project—increasing the number of sensors, such as adding optical barriers, with the goal of increasing the robustness of the body weight measurements and access to the behavior data gathered from the colony. Moreover, the authors are currently working on integrating the data gathered from the nests into the LifeWatch infrastructure.

## Figures and Tables

**Table 1.** Connection weight of the proposed MLP.

	**Neuron *N_2,1_***	**Neuron *N_2,2_***	**Neuron *N_2,3_***	**Neuron *N_3,1_***
Bias	−0.967833	−0.247893	3.395910	−0,778603
From *N_i-1,1_*	1.202640	2.025155	−2.773383	2,357087
From *N_i-1,2_*	−0.059006	0.280633	0.211419	3,643371
From *N_i-1,3_*	−0.419412	1.299244	−0.838220	−4,810658
From *N_i-1,4_*	−0.442465	0.771556	−0.075977	-
From *N_i-1,5_*	0.964739	2.033089	0.465052	-
From *N_i-1,6_*	−0.226521	0.920540	−0.065647	-
From *N_i-1,6_*	0.288798	2.659565	−0.522707	-
From *N_i-1,8_*	0.210606	0.844355	0.106924	-
From *N_i-1,9_*	0.759511	1.367401	0.129165	-

**Table 2.** Analysis of the database.

**Caption**	**Value**
Weight measurements	2,583,565
Number of patterns	51,517
Patterns with stable weights	7,856
Average pattern time	23.18 seconds
Days of test	399 days

**Table 3.** Analysis of the database.

**Caption**	**Energy (J)**
Without data aggregation	255.3
With data aggregation	0.608

**Table 4.** Weight accuracy on the basis of sporadic individual captures and the closest in time value offered by the automatic weighing system.

**Manual Weight/g**	**Date Manual Weight**	**Estimated Weight/g**	**Date Estimated Weight**	**Accuracy/%**	**PIT Code**
155	2010/06/14 12:35:51	154.471	2010/06/14 12:48:07	99.66	5103664
142	2010/06/21 12:21:21	144.078	2010/06/21 12:07:26	98.54	5103664
120	2010/06/21 13:00:15	118.045	2010/06/21 12:48:57	98.37	5104345
153	2010/06/14 14:52:01	154.716	2010/06/14 14:19:57	98.88	5103978
143	2010/06/21 11:57:35	144.661	2010/06/21 15:21:53	98.84	5103978
